# The views of pregnant women in New Zealand on vaginal seeding: a mixed-methods study

**DOI:** 10.1186/s12884-020-03500-y

**Published:** 2021-01-12

**Authors:** Éadaoin M. Butler, Abigail J. Reynolds, José G. B. Derraik, Brooke C. Wilson, Wayne S. Cutfield, Celia P. Grigg

**Affiliations:** 1 A Better Start – National Science Challenge, Auckland, New Zealand; 2 Liggins Institute, University of Auckland, Private Bag, Auckland, 92019 New Zealand; 3 Dartmouth College, Hanover, NH USA; 4 Department of Women’s and Children’s Health, Uppsala University, Uppsala, Sweden; 5 Children’s Hospital, Zhejiang University School of Medicine, Hangzhou, China

**Keywords:** Microbiome, Beliefs, Understanding, Mothers, Pregnancy, Caesarean section

## Abstract

**Background:**

Vaginal seeding is the administration of maternal vaginal bacteria to babies following birth by caesarean section (CS), intended to mimic the microbial exposure that occurs during vaginal birth. Appropriate development of the infant gut microbiome assists early immune development and might help reduce the risk of certain health conditions later in life, such as obesity and asthma. We aimed to explore the views of pregnant women on this practice.

**Methods:**

We conducted a sequential mixed-methods study on the views of pregnant women in New Zealand (NZ) on vaginal seeding. Phase one: brief semi-structured interviews with pregnant women participating in a clinical trial of vaginal seeding (*n* = 15); and phase two: online questionnaire of pregnant women throughout NZ (not in the trial) (*n* = 264). Reflexive thematic analysis was applied to interview and open-ended questionnaire data. Closed-ended questionnaire responses were analysed using descriptive statistics.

**Results:**

Six themes were produced through analysis of the open-ended data: “seeding replicates a natural process”, “microbiome is in the media”, “seeding may have potential benefits”, “seeking validation by a maternity caregiver”, “seeding could help reduce CS guilt”, and “the unknowns of seeding”. The idea that vaginal seeding replicates a natural process was suggested by some as an explanation to help overcome any initial negative perceptions of it. Many considered vaginal seeding to have potential benefit for the gut microbiome, while comparatively fewer considered it to be potentially beneficial for specific conditions such as obesity. Just under 30% of questionnaire respondents (*n* = 78; 29.5%) had prior knowledge of vaginal seeding, while most (*n* = 133; 82.6%) had an initially positive or neutral reaction to it. Few respondents changed their initial views on the practice after reading provided evidence-based information (*n* = 60; 22.7%), but of those who did, most became more positive (*n* = 51; 86.4%).

**Conclusions:**

Given its apparent acceptability, and if shown to be safe and effective for the prevention of early childhood obesity, vaginal seeding could be a non-stigmatising approach to prevention of this condition among children born by CS. Our findings also highlight the importance of lead maternity carers in NZ remaining current in their knowledge of vaginal seeding research.

**Supplementary Information:**

The online version contains supplementary material available at 10.1186/s12884-020-03500-y.

## Background

There has been a worldwide increase in the rate of caesarean section (CS) [1], a surgical procedure intended to provide a safer alternative to vaginal birth when clinically indicated. In New Zealand (NZ), approximately one in four babies are born by CS [2]. However, birth by CS has also been associated with increased risk of adverse health outcomes in the long-term, including asthma [3], type 1 diabetes [4], coeliac disease [5], and childhood obesity [6]. The microbiome of infants born by caesarean section differs from those of infants born vaginally [7, 8]. This may be due to a lack of exposure to vaginal bacteria during birth, a critical period of transition and microbial colonisation, and potentially explains the increased risk of adverse health outcomes associated with birth by CS [9].

Vaginal seeding is a relatively new practice, whereby maternal vaginal bacteria are artificially administered to babies following birth by CS [10]. This is intended to mimic the natural process of maternal microbial exposure that occurs during a vaginal birth [11]. However, there exists only one published study that tested vaginal seeding [11]. Published in 2016, this very small pilot study showed that the skin, oral, and anal microbiomes of four seeded CS-born babies were more similar to those of seven vaginal-born babies than seven non-seeded CS-born babies [11]. The study did not evaluate the gut microbiome, with no faecal samples collected. While promising, these results are far from conclusive. Our group is currently conducting the Early COlonisation with Bacteria After Birth (ECOBABe) study, assessing the effectiveness of vaginal seeding in establishing the gut microbiome in CS-born babies [12], whilst other trials are ongoing elsewhere [13–15].

Despite the lack of evidence for the effectiveness of vaginal seeding, the practice has been widely publicised by the lay press [16]. In particular, the award-winning 2014 documentary “Microbirth” which focused on the normal physiological process of microbiome establishment [17], also featured the lead author of the previously mentioned seeding study, discussing that research. This widespread publication, as well as concerns regarding the potential transmission of Group B *Streptococcus*, prompted the American College of Obstetricians and Gynecologists to release a committee opinion advising against performing vaginal seeding outside the context of a research study, at least until data regarding safety and benefit are available [16]. In fact, the practice has sparked lively debate in the medical/scientific literature [10, 16, 18–21], including a journal editorial titled “Perplexing perinatal practices” [22]. However, while there are numerous lay sources of information on the topic, as well as more articles sharing the perspectives of clinicians and scientists than seeded babies in the original study, the views of pregnant women are yet to be investigated.

This lack of investigation of maternal views is particularly important given that Western society, for the latter part of the nineteenth century, has been encouraged to view bacteria as something to be eliminated rather than embraced [23]. Despite advancements in our knowledge of the beneficial properties of many bacteria, numerous products are marketed to the general public claiming to kill bacteria in order to prevent sickness, thus further reinforcing the idea all microorganisms are harmful [24]. While several studies have examined patient perceptions of bacterial transfer as a treatment, they have focused on faecal microbiome transplantation in adults with severe and/or chronic illnesses (recurring *Clostridium difficile* infection or ulcerative colitis) [25–28]. Thus, these studies do not necessarily reflect the views of pregnant women on vaginal seeding, where the benefit is a potential reduction in their child’s risk of future obesity or other conditions, rather than immediate relief from a chronic or acute illness. If the ongoing clinical trials prove vaginal seeding to be an effective treatment, it would be valuable to understand maternal views, so that healthcare practitioners can best communicate any benefits or risks with mothers.

Therefore, in order to address this gap, we conducted a novel, exploratory, mixed-methods study of the views of pregnant women on vaginal seeding. In particular, we sought to understand their initial perception of the practice, their current views of it, and the factors that might motivate them to undertake vaginal seeding. Additionally, we aimed to understand if the provision of detailed information regarding vaginal seeding would change the initial perception of questionnaire respondents on the practice.

## Methods

### Design

**Fig. 1:**
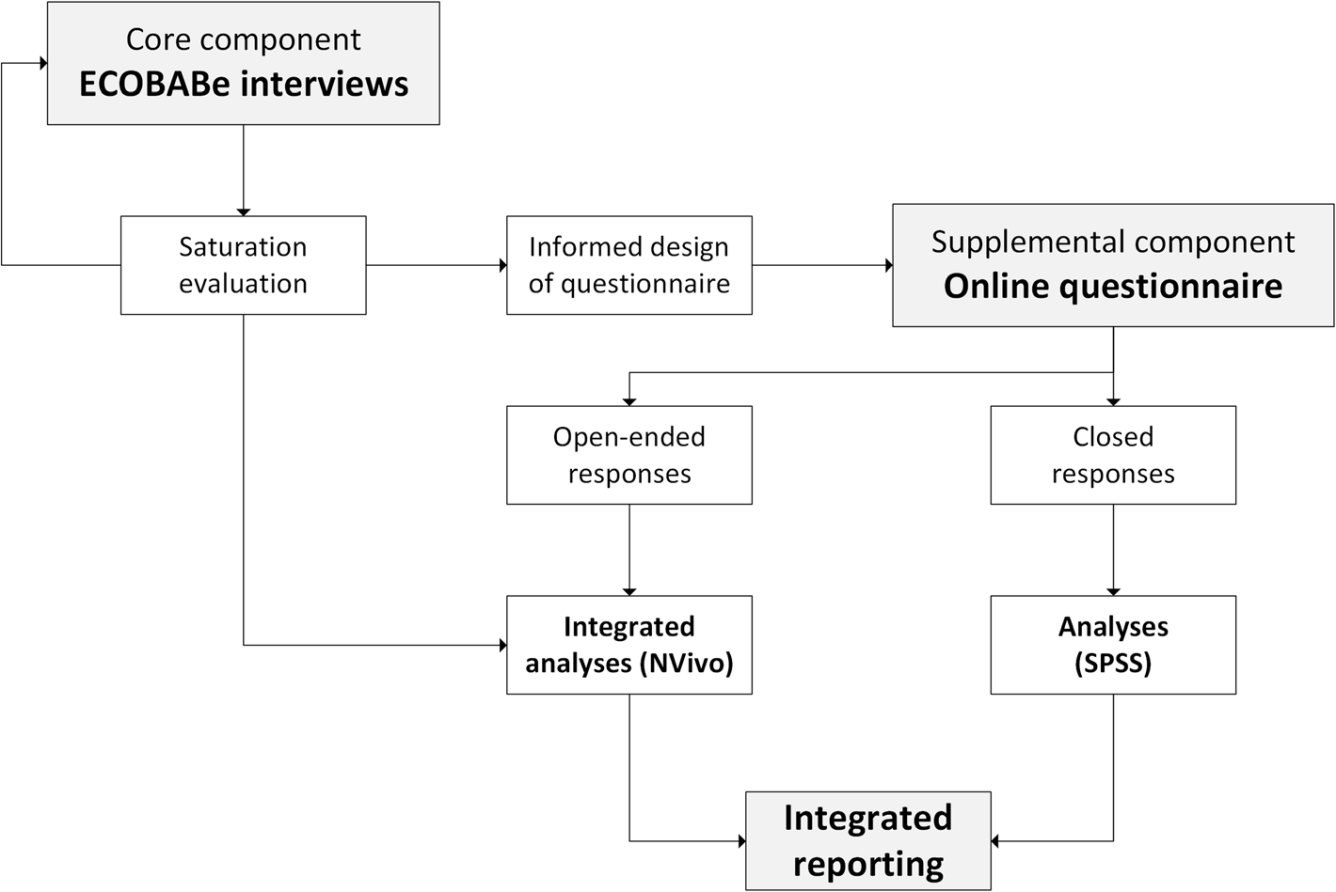
Flow-chart outlining the study design

The study design is sequential mixed-methods, with phase one consisting of interviews with women in the CS group of the ECOBABe study and phase two consisting of an online questionnaire of pregnant women throughout NZ, not participating in the ECOBABe study. The questionnaire was designed with the content and preliminary analysis of the interviews in mind. Thus, the two phases of the study were sequential with: separate samples and data collection methods, integrated analysis of interviews and open-ended questionnaire responses, and integrated reporting of all findings (Fig. 1). Ethical approval for phase one was given by the Northern A Health and Disability Ethics Committee, New Zealand and for phase two by the University of Auckland Human Participants Ethics Committee.

### Phase one: interviews

We conduced brief, face-to-face, semi-structured interviews with some women participating in the CS group of the ECOBABe trial. The interview guide (Supplementary File 1) was developed by the research team, based on observations of women’s responses to the concept of vaginal seeding during recruitment for the overall ECOBABe trial. Participation in the interviews was an optional add-on for these women, where a woman’s decision to participate (or not) did not affect her status or treatment in the trial. Further, women did not receive any additional incentive to participate in interviews, beyond what was already offered in the ECOBABe trial (i.e. reimbursement for travel costs).

The interviews took place at the Liggins Institute Clinical Research Unit, just prior to women’s health screening tests for eligibility to further participate in the ECOBABe trial [12]. Interviews were audio-recorded and lasted up to 10 min, conducted by one of two researchers (ÉMB or CPG).

### Phase two: online questionnaire

The inclusion criteria for the online questionnaire (Supplementary File 2) were: pregnant women, aged ≥18 years, resident in NZ, and not participating in the ECOBABe study. We excluded participants from the ECOBABe study on the basis that they would have received comprehensive information from the research team, and would therefore have been biased. We aimed to assess the views of a broader group who had either never heard of it, or had previously heard of it but not through the research team. The questionnaire was constructed and delivered using an online platform (Qualtrics Labs Inc., Provo, UT, USA), and was distributed in January and February 2020 via social media (Facebook and Twitter). Respondents did not receive any incentive for participation.

The questionnaire consisted of 13–18 questions (including some that were response-dependent), and took approximately 10 min to complete. Questions were a mixture of yes/no, Likert scales, multiple choice, and open-ended. Questions differed according to whether or not the respondent had heard of vaginal seeding prior to participating in our questionnaire. Respondents without prior knowledge of the practice were initially provided with this brief explanation: *“Vaginal seeding involves giving babies born by caesarean section some of their mother’s vaginal bacteria just after birth”.* This was followed by more detailed evidence-based information on the practice, which was also presented to respondents with prior knowledge. This was because we wanted to assess the impact of the provision of this evidence-based information on the respondent’s initial perception of the practice and explore if any change in perception differed according to whether or not respondents had heard of vaginal seeding before participating in our questionnaire. We also collected demographic information (age, ethnicity, and education), as well as the respondent’s current planned birth mode (vaginal, CS, or unsure).

### Analyses

The audio-recorded interview files were de-identified prior to professional transcription and the files were uploaded to NVivo 12 (QSR International Pty Ltd) for analysis. Reflexive thematic analysis [29, 30] was applied to the interview data and open-ended questionnaire responses. Collaborative inductive coding of data occurred between three investigators (ÉMB, CPG, and AJR), with similarities and contrasts between interview and open-ended questionnaire responses identified. Candidate themes were revised multiple times throughout the analytical process. Through discussion, ÉMB and CPG reached final consensus regarding themes for reporting, based on their relevance to the research questions. Quotes are reported in italics using anonymised participant codes, where codes beginning in “I” and “Q” represent interviewees and questionnaire respondents, respectively.

All remaining questionnaire data and sociodemographic statistics for interviewees were analysed using SPSS v26 (IBM Corp, Armonk, USA). Descriptive statistics were provided for closed questionnaire questions. Ethnicity was defined using a hierarchical system of classification, with all participants assigned a single ethnicity in the following order of priority: Māori, Pacific, Asian, and European (the latter including NZ European and other European/Caucasian) [31]. We did not have any participants who were classified as ‘MELAA’ (Middle Eastern, Latin American, or African) or ‘Other’. Figures were prepared using GraphPad Prism v8.2 (GraphPad Software Inc., San Diego, CA, USA).

Results are presented in the following order: 1) participant demographics, 2) thematic results generated from interviews and open-ended questionnaire questions (as well as relevant closed questionnaire questions), and 3) results regarding the impact of the provision of evidence-based information about vaginal seeding on questionnaire respondents’ views of the practice.

## Results

### Participant demographics

Table 1 shows the sociodemographic characteristics of the 15 interviewees and 264 questionnaire respondents. The majority of participants were of European ethnicity and highly educated, although there was a lesser proportion of university-educated questionnaire respondents compared to interviewees (Table 1). Additionally, there was a greater proportion of interviewees who were ≥ 35 years of age than questionnaire respondents (Table 1). Most questionnaire respondents were planning a vaginal birth (80.6%) and had not previously heard of vaginal seeding (70.5%) (Table 1).

**Table 1:** Characteristics of participants

	Interviewees	Questionnaire respondents
**n**	15	264^a^
**Ethnicity**		
European	11 (73.3%)	223 (84.5%)
Māori	2 (13.3%)	33 (12.5%)
Pacific	1 (6.7%)	4 (1.5%)
Asian	1 (6.7%)	4 (1.5%)
**Highest education**		
No qualification	nil	7 (2.7%)
High-school qualification	nil	27 (10.3%)
Post-school vocational qualification	3 (20.0%)	60 (22.8%)
University degree	12 (80.0%)	169 (64.3%)
**Participant age (years)**		
18 to ≤25	nil	16 (6.1%)
26 to ≤35	6 (40.0%)	198 (75.9%)
≥ 36	9 (60.0%)	47 (18.0%)
**Planned birth mode**		
Elective caesarean section	15 (100%)	28 (10.6%)
Vaginal	nil	212 (80.6%)
Unsure	nil	23 (8.7%)
**Had heard of vaginal seeding**		
Yes	15 (100%)	78 (29.5%)
No	nil	186 (70.5%)

Data are *n*(%)

^a^Not all 264 included respondents answered all questions; *n* (%) for individual categories were: ethnicity (264; 100%), education (263; 99.6%), participant age (261; 98.9%) and planned birth mode (263; 99.6%)

### Themes

We generated six distinct themes from the interview transcripts and open-ended questionnaire data: “seeding replicates a natural process”, “microbiome is in the media”, “seeding may have potential benefits”, “seeking validation by a maternity caregiver”, “seeding could help reduce CS guilt”, and “the unknowns of seeding”.

### Seeding replicates a natural process

This theme was very strong among both interviewees and questionnaire respondents. Many commented on how they believed seeding to be logical or something that *“brings a little more naturalness to the birth”* (I-016). In fact, almost all interviewees expressed this view at some point during their interview. For many interviewees, the idea that seeding replicates something natural helped overcome any initial adverse reactions to the concept (experienced by themselves or others), as explained by this participant:

Interviewer: *“Did it make sense when you first heard it, like straight away?”*

Participant: *“Yes, yeah.*”

Interviewer: *“Yeah?”*

Participant: *“Yeah I mean there’s a part of you that’s always like “oh that’s a bit gross”…but then you’re like “oh no, it’s actually really natural and it’s fine”.”* (I-025)

Numerous questionnaire respondents shared similar views; for example, *“The thought of deliberately swabbing baby with vaginal secretions is not a really pleasant thought - however, I know from a non-emotional perspective I know it somewhat replicates the transfer of vaginal flora to the baby during vaginal delivery so absolutely not against it!”* (Q-191).

### Microbiome is in the media

This theme encompassed a sense that discussions about the microbiome and its relevance to babies was *“something that’s sort of out in the media a lot”* (I-028). One interviewee distinguished between the presence of information about the gut microbiome and vaginal seeding itself:

*“It’s very much in the media at the moment. Not vaginal seeding as such but gut biome and…*

*stuff like that. If you’re on Facebook it’s just all over it, yeah.”* (I-024)

**Fig. 2:**
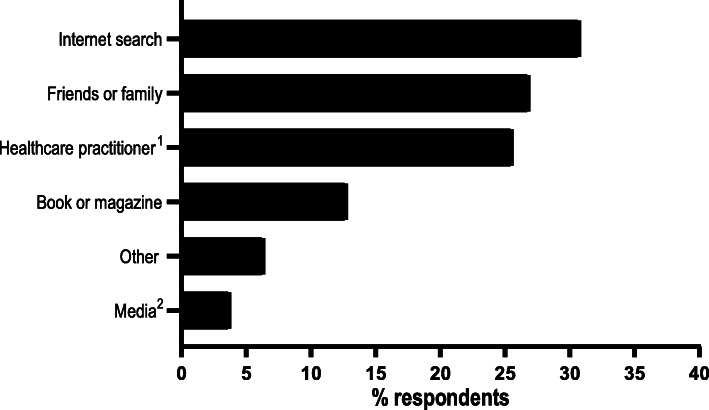
Frequency of responses to “Where did you hear about vaginal seeding?” by questionnaire respondents who had prior knowledge of vaginal seeding (*n* = 78).^1^Includes respondents who reported hearing about vaginal seeding in a healthcare setting (e.g. clinic waiting room or antenatal class).^2^Includes respondents who reported hearing about vaginal seeding through traditional media (e.g. radio or TV) or social media (e.g. Facebook or Twitter). Respondents could select multiple options

In support of the above, Fig. 2 shows that very few of our online questionnaire respondents who had previous knowledge of seeding reported hearing about it from the media (either traditional or social, *n* = 3; 3.8%). Instead, most of these women had heard about seeding via an internet search (*n* = 24; 30.8%), from friends or family (*n* = 21; 26.9%), or from a healthcare practitioner (*n* = 20; 25.6%). Nonetheless, two of the 15 interviewees did report hearing about seeding from a television documentary, while others mentioned that they had become aware of CS-born babies missing out on contact with vaginal bacteria through the media.

### Seeding may have potential benefits

**Fig. 3:**
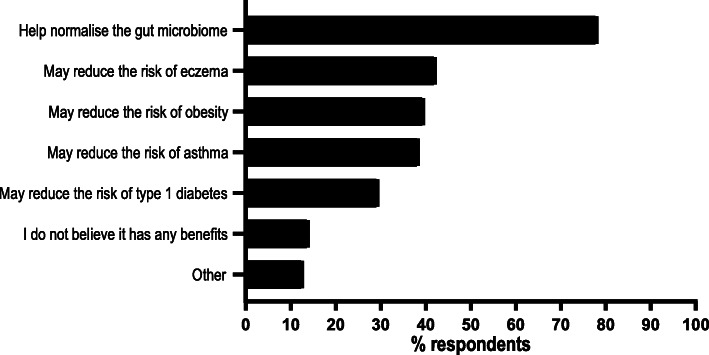
Frequency of responses to “What do you believe the potential benefits [of vaginal seeding] to be, if any?” by questionnaire respondents who had prior knowledge of vaginal seeding (*n* = 78). Respondents could select multiple options

Many participants (interviewees and questionnaire respondents) felt there were at least some potential benefits to vaginal seeding, often citing a general benefit to the gut microbiome. In fact, “Help normalise the gut microbiome” was selected as a potential benefit of seeding by almost 80% (*n* = 61; 78.2%) of questionnaire respondents who had previously heard of vaginal seeding, whereas less than half believed it to have any benefit for specific conditions like obesity (Fig. 3). During interviews and in open-ended questionnaire responses, some women cited personal experience as evidence for potential benefits, such as in the below interview exchange with one ECOBABe participant:

Interviewer: *“What were your thoughts when you heard for the first time, about the idea of giving vaginal bacteria to babies born by caesarean section?”*

Participant: *“I thought it was a really good idea, just because I had my first child and he was done by c-section, and I just felt like he didn’t receive the same type of benefits as if he was just born vaginally.”*

Interviewer: *“Yeah okay. What kind of benefits?”*

Participant: *“Well for example right now I feel like he’s got eczema, he’s got things like his immune’s not that great.”* (I-029)

Similarly, one questionnaire respondent shared how her views of seeding were shaped by her own health condition, as well as that of her sister’s: *“Having been born by caesarean and having what I believe was slight childhood obesity. My sister was also born by caesarean and developed type 1 diabetes as an adult,”* (Q-109).

Some participants also expressed a view that seeding was a ‘low risk, no harm’ practice. For these women, regardless of whether they felt seeding could confer any specific benefit or not, they viewed it as something that was *“worth a shot for whatever it may do”* (Q-013). Interviewee I-026 put it as: *“if it makes a difference, good, and if it doesn’t make any difference, it’s not like anything’s changed or been lost”.* Some of the interviewees expressed this view in the context of feeling safe as a participant in a research study; for example, *“I was excited for there be like a study about it and be more controlled rather than having to do it myself,”* (I-015).

In contrast to the above, a number of questionnaire respondents expressed sceptical views about the legitimacy of seeding and its potential to provide any health benefits. For example, one woman stated: *“sounds like pseudoscience, and nasty pseudoscience at that”* (Q-153), while another said: *“based on the name ‘vaginal seeding’ alone my instinct was that it sounded dodgy…something alternative and not backed up by evidence. When I found out what it was, I thought ‘ok, maybe there’s something in that, but I still want to see a published, peer reviewed, clinical trial...’!”* (Q-242). These views were not expressed by interviewees, which is unsurprising given they had volunteered to participate in a vaginal seeding clinical trial.

### Seeking validation by a maternity caregiver

When asked what encouraged them to participate in the ECOBABe study, several interviewees reported having spoken to their lead maternity carer (LMC) about seeding or the study itself. Most had initiated the discussion, although some LMCs had raised the topic first. These interviewees seemed to value their LMC’s support with participation in the study and seeding in general:

*“It was I guess after finding out that we had to have a c-section, and then somebody else then said it at our antenatal class. “Oh that rings a bell, I’ve heard about that before”. And then when I went to my obstetrician and I asked him, and I said “look I’ve heard about this thing, is it, you know what’s it all about?” And he said that he backs it.”* (I-044)

*“Well I asked Doctor (name) if he could do it* [seeding]*. And then he said “oh no, but there’s a trial, there’s this research out at Liggins Institute that do it”.”* (I-029)

Likewise, one questionnaire respondent shared how she planned to ask her LMC about seeding if she decided on an elective CS: *“I had an emergency c-section with my first child and so didn’t have a chance to implement it - will definitely be asking my LMC about it if I decide on an elective this time around.”* (Q-041). Another said she would not perform seeding *“until the evidence was there and the medical profession was supporting it”* (Q-242).

**Fig. 4:**
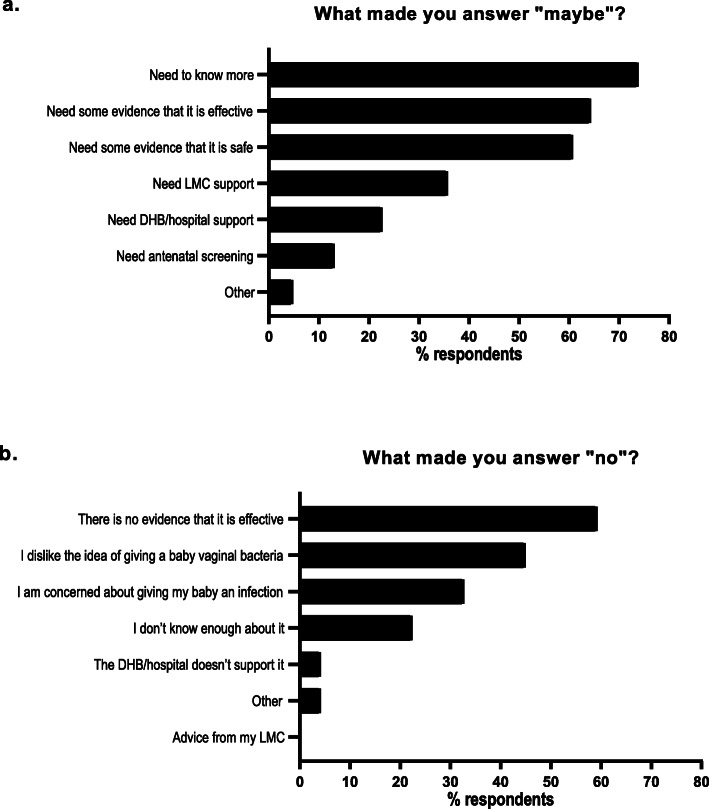
Frequency of reasons why questionnaire respondents selected “maybe” (*n* = 84) or “no” (*n* = 49) when asked “Given what you know about vaginal seeding, would you consider doing it if you were to have a caesarean section?” DHB, district health board; LMC, lead maternity carer. Respondents could select multiple options

When asked if they would perform vaginal seeding if they were to have a CS (based on any prior information and/or the information we provided), approximately half of questionnaire respondents said “yes” (*n* = 131; 49.6%), just under one-third “maybe” (*n* = 84; 31.8%), and less than 20% “no” (*n* = 49; 18.6%). Of the respondents who selected “maybe”, about one-third (*n* = 30; 35.7%) said they would require LMC support, while one-fifth said they would require District Health Board (DHB) or hospital support (*n* = 19; 22.6%) (Fig. 4a). However, needing more information (*n* = 62; 73.8%), evidence regarding effectiveness (*n* = 54; 64.3%), and evidence regarding safety (*n* = 51; 60.7%) were the three most frequent reasons for these women selecting “maybe” (Fig. 4a). Of those who responded “no” when asked if they would perform vaginal seeding, lack of evidence regarding effectiveness (*n* = 29; 59.2%), a dislike of giving a baby vaginal bacteria (*n* = 22; 44.9%), and concern regarding giving their baby an infection (*n* = 16; 32.7%) were the top three reasons for doing so (Fig. 4b). Very few women (n = 2; 4.1%) selected “the DHB/hospital doesn’t support it”, while none selected “advice from my LMC” (Fig. 4b).

### Seeding could help reduce CS guilt

Some women shared how they believed that seeding could be a way to help alleviate feelings of guilt experienced by women undergoing CS, by helping “*provide the baby with the benefits* [of vaginal birth] *in another way”* (I-007). When asked what encouraged her to join the ECOBABe study, one interviewee responded:

Participant: *“I guess just that I don’t have to feel as guilty about having a c-section…like at least I know I’ve done everything I can do then.”*

Interviewer: *“Yeah, yeah...”*

Participant: *“I know I’ve done everything.”* (I-026)

This theme was less apparent among questionnaire respondents (notably, we did not ask any specific questions regarding CS guilt). Nonetheless, one respondent explained how she felt positive about seeding because she wanted her *“baby to have the best birth experience possible”*, and if she needed to have a CS, she *“was heartened to know* [she] *had ways to mitigate some negatives”* (Q-209)*.* Conversely, one questionnaire respondent felt that seeding could add further to CS guilt, stating: *“I think it has little to no benefit and is one more thing to guilt c-sec mothers with”* (Q-045).

### The unknowns of seeding

This theme was unique to questionnaire respondents, many of whom expressed the view that there was a lot more for them (and researchers) to learn about vaginal seeding. For some this was on a mechanistic level, reporting they would “maybe” perform vaginal seeding because they would need to know how the procedure itself was performed. However, many of the comments related to this theme were responses to reading the evidence-based information about vaginal seeding presented in the questionnaire. For instance, some respondents were previously unaware of the limitations of current seeding research; one respondent said she learned *“that there is actually so little research about it”* (Q-058). Furthermore, a few respondents reported they had been previously unaware of long-term health associated with birth by CS: *“I wasn’t aware of increased risk of asthma, diabetes and eczema with caesarean births”* (Q-195)*,* or *“the risk of infection”* transmission through seeding (Q-124).

### Impact of evidence-based information on questionnaire respondents perceptions of vaginal seeding

**Fig. 5:**
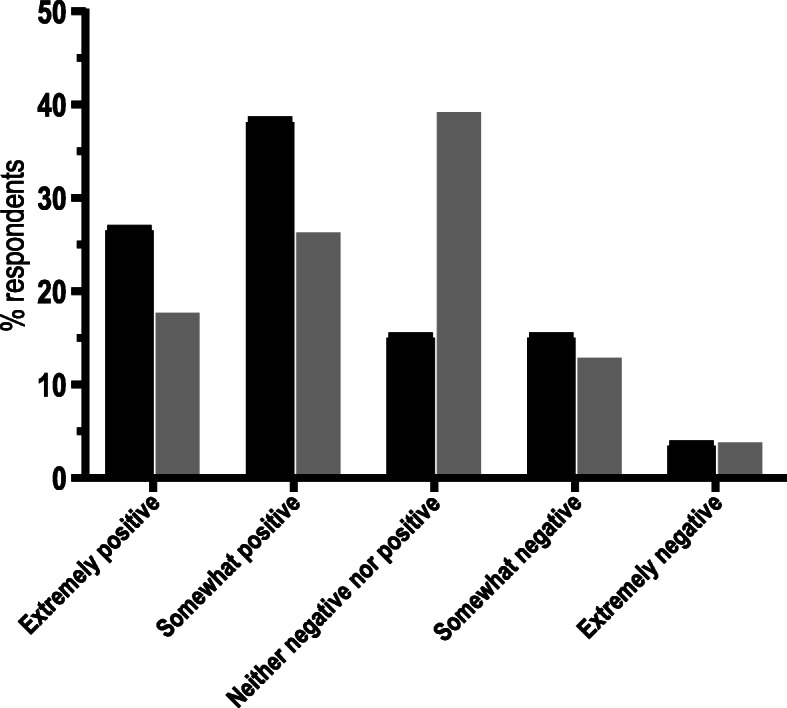
Initial perception of vaginal seeding for questionnaire respondents who had previous knowledge of it (black bars; n = 78) and those who did not (grey bars; *n* = 186). Respondents who had not previously heard of vaginal seeding were given a one sentence explantion of the practice: *“Vaginal seeding involves giving babies born by caesarean section some of their mother’s vaginal bacteria just after birth”*

**Fig. 6:**
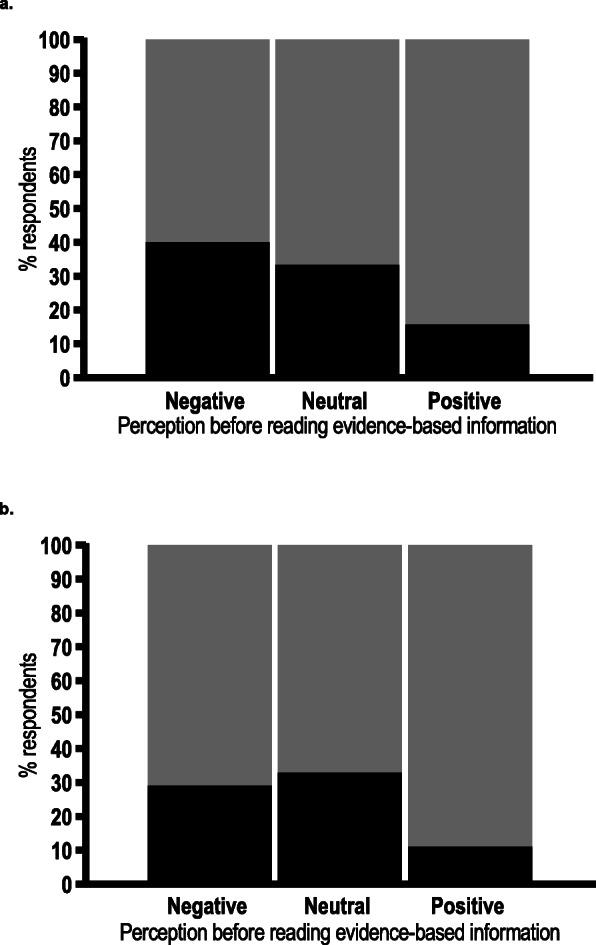
Proportion of respondents’ changing their initial perceptions of vaginal seeding after reading evidence-based information provided in the questionnaire. **a** respondents who had heard of vaginal seeding before (*n* = 78). **b** respondents who had not heard of vaginal seeding before (*n* = 186). Black represents respondents who changed their views, grey represents respondents who did not

**Fig. 7:**
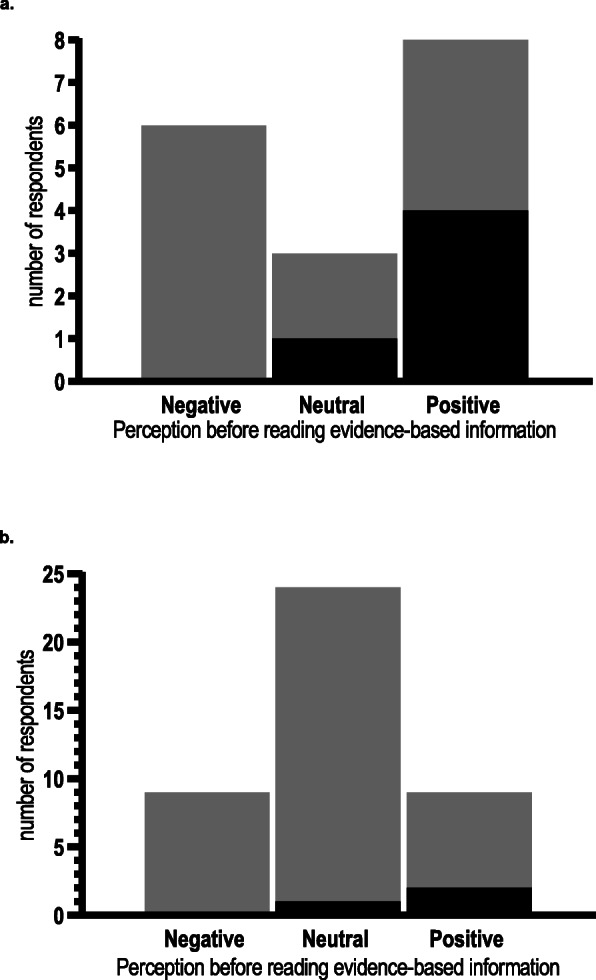
Direction of change of respondents’ perceptions of vaginal seeding for those who changed their views following reading evidence-based information provided in the questionnaire. **a** respondents who had heard of vaginal seeding before and changed their views (*n* = 17).^1^ **b** respondents who had not heard of vaginal seeding before and changed their views (*n* = 42). Black represents respondents who became more negative, grey represents respondents who became more positive. ^1^18 respondents with prior knowledge of vaginal seeding reported changing their views following reading the provided evidence-based information, but only 17 provided the direction of their change of view

Over 80% of questionnaire respondents (*n* = 133; 82.6%) reported a positive or neutral initial reaction to vaginal seeding. A greater proportion of respondents who had heard of the practice prior to participating in the questionnaire had a positive initial reaction (*n* = 51; 65.4%), compared to those who had just read our brief one-sentence explanation (*n* = 82; 44.1%) (Fig. 5). Furthermore, a greater proportion of respondents without prior knowledge selected “neither negative nor positive” (*n* = 73; 39.2%), than those with prior knowledge (*n* = 12; 15.4%) (Fig. 5). Only 23.1% (*n* = 18) of respondents with prior knowledge of seeding changed their views following reading the evidence-based information we provided (Fig. 6a), while a similar proportion of women (*n* = 42; 22.6%) who did not have prior knowledge changed their views (Fig. 6b). A greater proportion of respondents who initially felt negative or neutral about the practice changed their views than those who initially felt positive (Fig. 6a and b). Of those who did change their views, the majority became more positive (*n* = 51; 86.4%), although half of the eight women with prior knowledge who had initially felt positive, changed to a more negative view of the practice (Fig. 7a and b).

## Discussion

Our thematic analysis of open-ended data (collected in both interview and questionnaire formats), produced six themes representing the views of some pregnant women in NZ on vaginal seeding. The majority of questionnaire respondents felt positive or neutral about the practice, regardless of whether or not they had heard of it before, while only a small proportion changed their views following reading the provided evidence-based information. But, most of those who did change their views became more positive. Our results may be of interest to those researching microbiome interventions for health conditions and lead maternity carers (LMCs).

Most participating women viewed seeding as replicating a natural process, which some proposed was a way of explaining the concept to increase its acceptability. For a few, it was also suggested as a way of reducing guilt experienced by women planning a CS birth. Most participants did not seem to believe vaginal bacteria should be avoided in this context (although almost 45% of questionnaire respondents who said they would not perform it cited a dislike of giving a baby vaginal bacteria as a reason why). Many participants considered vaginal seeding to have a potential benefit for the gut microbiome, while comparatively fewer considered it to have the potential to reduce the risk of specific health conditions such as obesity. Based on currently available evidence, many of our participants were technically correct in their responses; it is currently unknown if vaginal seeding reduces the risk of any specific health conditions, while only one very small study suggests it may shift the microbiome of infants born by caesarean section to more closely reflect that of infants born vaginally [11]. Larger and long-term studies are currently ongoing to assess the impact of seeding, not only on the gut microbiome [12–15], but also on childhood obesity [13, 14], allergic diseases [13, 15], and asthma [15]. Notably, childhood obesity is a health condition that is particularly subject to stigma; in fact, healthcare professionals report not raising the topic for fear of damaging their relationships with families [32]. Given the association between obesity and imbalance of the gut microbiome [33], interventions such as vaginal seeding - which was largely viewed in a positive or neutral light by our questionnaire respondents - could have the potential to help with its prevention, without placing primary focus on the condition itself.

The provision of evidence-based information about vaginal seeding (which included an explanation of the practice as an attempt to “mimic the contact a baby would normally have with their mother’s vaginal bacteria during birth”) did little to change the initial perception of questionnaire respondents, even for those who had based it on a brief one-sentence explanation. Furthermore, only about 30% of questionnaire respondents with an initially negative view of vaginal seeding changed their views. For those whose views did change, they mostly became more positive. A few questionnaire respondents with prior knowledge of vaginal seeding stated that they had been previously unaware of any potential risks associated with the practice or the lack of research on it. Moreover, some participants felt vaginal seeding was worth performing even in the absence of any tangible benefit, although many interviewees framed this in the context of feeling safer in a research study. Several interviewees and a few questionnaire respondents reported communication with their LMC regarding participation in the ECOBABe study (interviewees only) or vaginal seeding in general. These participants seemed to value their LMCs perspective on the topic. As previously noted, current evidence for any benefit of vaginal seeding is limited and largely theoretical, but we are aware of one case where herpes simplex was transmitted to a baby following vaginal seeding [34]. Currently available clinical guidelines on vaginal seeding are few and conservative [16, 20], but this may change in light of new evidence produced by ongoing trials. It is therefore important that LMCs remain up-to-date in their knowledge of vaginal seeding to inform risk-benefit conversations with women, especially as almost 30% of questionnaire respondents had already heard of the practice prior to starting the questionnaire.

### Limitations and strengths

In what we believe to be the first study to explore women’s views on vaginal seeding, well-educated women of European (Caucasian) ethnicity were over-represented, such that we cannot readily extrapolate our results to women of lesser education or other ethnicities. Notably, Māori and Pacific women were under-represented. One ECOBABe mother alluded to potential cultural differences in acceptance of vaginal seeding, mentioning that “[as] *a Pacific Islander, you don’t even talk about that*” (M-016), but we did not have enough data on this to create a distinct theme. The applicability of ‘saturation’ to thematic analysis has recently been questioned [35], and it may be that had we recruited more Māori or Pacific women, such a theme would have been viable. Future research should seek to explore the views of Māori and Pacific women on this topic, especially if it is shown to be an effective treatment for the prevention of early childhood obesity. Particularly since Māori and Pacific children experience disproportionately higher rates of this compared to peers of other ethnic backgrounds [36]. Our questionnaire was also limited by the relatively small proportion of respondents planning an elective CS birth (about 10%). While this is comparable to the national rate of birth by elective CS in 2017 (12.6%) [2], it is possible that a study focussing only on women planning CS births would have different findings. Nonetheless, a key strength of our study is the fact that approximately two-thirds of women had no prior knowledge of vaginal seeding before participating, thus it is unlikely that our results were affected by selection bias. Additionally, our use of independent, followed by collaborative, coding by three researchers lends support to the validity of our themes.

## Conclusions

Vaginal seeding was viewed in a positive or neutral light by most pregnant women participating in our study, some of whom felt that explaining it as replicating a natural process could overcome any initial negative perceptions of the practice. Many considered it to have a benefit for the gut microbiome, while fewer considered it to have benefit for specific health conditions. If proven to be safe and effective for the prevention of early childhood obesity, vaginal seeding may have the potential to be a non-stigmatising approach to reducing the prevalence of this condition among children born by CS. Our findings also highlight the importance of LMCs remaining current in their knowledge of vaginal seeding research.

## Supplementary Information

**Additional file 1: Supplementary File 1**: Interview schedule. **Supplementary File 2**: Online questionnaire content

## Data Availability

The data generated and/or analysed during the current study are not publicly available as permission to grant public availability of the dataset was not included in our ethics approvals for this study.

## Abbreviations

CS: Caesarean section
DHB: District Health Board
ECOBABe: Early COlonisation with Bacteria After Birth
LMC: Lead maternity carer
MELAA: Middle Eastern, Latin American, or African
NZ: New Zealand
