# Supplementary material for: The views of pregnant women in New Zealand on vaginal seeding: a mixed-methods study
Source: BMC Pregnancy Childbirth. 2021 Jan 12;21:49. doi: 10.1186/s12884-020-03500-y (PMC7802193; doi:10.1186/s12884-020-03500-y)
Supplement: Supplementary file 1 — Additional file 1: Supplementary File 1: Interview schedule. Supplementary File 2: Online questionnaire content [file 12884_2020_3500_MOESM1_ESM.docx]

**Supplementary File 1: Interview schedule**

1. What were your thoughts when you heard for the first time about the idea of giving vaginal bacteria to babies born by caesarean section?
2. Before learning more about the ECOBABe study, would you have ever considered vaginal seeding for your baby?
3. What encouraged you to participate in the ECOBABe study?
4. What do you believe to be the potential benefits of vaginal seeding (if any)?
5. Do you have any concerns about the fact that your baby will receive vaginal seeding?
6. Have you told family and/or friends that you are participating in the trial, and if so, what were their responses?
7. How would you explain the reasons for giving vaginal bacteria to a baby born by caesarean section to another pregnant woman?
8. Is there anything else you would like to add before we end the interview?

**Supplementary File 2: Online questionnaire content**

*Initial question:*

Have you heard of ‘vaginal seeding’ before?

| Yes |  |
| --- | --- |
| No |  |

*Question pathway for respondents who* ***had*** *previously heard of vaginal seeding.*

Q 1. Where did you hear about it (choose as many as apply)?

| Friend |  |
| --- | --- |
| Family |  |
| LMC |  |
| GP |  |
| Obstetrician |  |
| Internet search |  |
| Social media (eg Facebook, Twitter etc) |  |
| Traditional media (Radio, TV) |  |
| Book or magazine |  |
| Other (specify)_________________________________________________ |  |

Q 2. How positive or negative were your first thoughts about vaginal seeding?

| Extremely positive |  |
| --- | --- |
| Positive |  |
| Neither positive nor negative |  |
| Negative |  |
| Extremely negative |  |

Q2a. What made you choose that option?

|  |
| --- |

Q3. What do you believe the potential benefits to be, if any (tick as many as apply)?

| Help normalise the gut microbiome |  |
| --- | --- |
| May reduce risk of asthma |  |
| May reduce risk of Type 1 diabetes |  |
| May reduce risk of eczema |  |
| May reduce risk of obesity |  |
| I do not believe it has any benefits |  |
| Other (specify) ________________________________________________ |  |

*The below information was then shown to respondents.*

Vaginal seeding involves giving babies born by caesarean section some of their mother’s vaginal bacteria just after birth. This is to try and mimic the contact a baby would normally have with their mother’s vaginal bacteria during birth.

Research has shown that babies born by c-section have small increased risks of conditions like asthma, eczema, type 1 diabetes, and childhood obesity, even when the reasons for the caesarean section and other background factors are taken into account. Their gut microbiome (the collection of bacteria and other organisms that live in their gut) is also different to that of babies that were born vaginally. It is possible that this gut microbiome difference for c-section babies may be partly due to missed contact with vaginal bacteria during birth. In turn, the differences in gut microbiome may be partly responsible for the increased health risks seen for babies born by c-section. Vaginal seeding is a potential way of helping to make the gut microbiome of babies born by c-section look more like that of vaginally-born babies, and possibly reduce associated risks to their health. 

However, there is currently no research to show that it works, either in the short term by colonising the baby’s gut, or improving long term health of the child. Only one previous study of 4 seeded babies has been published. The researchers found a difference in the 4 babies’ skin, oral, and anal microbiome at 1 month (compared with the 7 unseeded babies), but they did not check their gut microbiome. Additionally, it is possible that vaginal seeding could place babies born by c-section at risk of infection (if appropriate screening tests are not performed beforehand).

Q4. Have you learned anything new or different from what you understood about it?

| Yes |  |
| --- | --- |
| No |  |

*If respondent selected “yes” to Q4, Q4a appeared.*

Q4a. Can you tell us what’s new or different?

|  |
| --- |

Q5. Have your views on vaginal seeding changed since you first heard about it?

| Yes |  |
| --- | --- |
| No |  |

*If respondent selected “yes” to Q5, Q5a appeared.*

Q5a. Have your views become more or less positive?

| Much more positive |  |
| --- | --- |
| A bit more positive |  |
| A bit more negative |  |
| Much more negative |  |

Q6. Given what you know about vaginal seeding, would you consider doing it if you were to have a caesarean section?

| Yes |  |
| --- | --- |
| Maybe |  |
| No |  |

*If respondent selected “maybe” to Q6, Q6a appeared.*

Q6a. What made you answer ‘Maybe’ (select all that apply)?

| Need to know more |  |
| --- | --- |
| Need LMC support |  |
| Need DHB/hospital support |  |
| Need antenatal screening |  |
| Need more evidence that it is safe |  |
| Need some evidence that it is effective |  |
| Other (specify) ________________________________________________ |  |
| Nothing, I would not do it |  |

*If respondent selected “no” to Q6, Q6b appeared.*

Q6b. What made you answer ‘No’?

| I dislike the idea of giving a baby vaginal bacteria |  |
| --- | --- |
| I don’t know enough about it |  |
| Advice from my LMC |  |
| The DHB/hospital doesn’t support it |  |
| I am concerned about giving my baby an infection |  |
| There is no evidence that it is effective |  |
| Other (specify) ________________________________________________ |  |

Q7. Is there anything else you’d like to tell us about your views on vaginal seeding?

| Yes |  |
| --- | --- |
| No |  |

*If respondent selected “yes” to Q7, Q7a appeared.*

Q7a. Please tell us about these thoughts or views

|  |
| --- |

*---------------------------------------------------------------------------------------------------------------------------*

*Question pathway for respondents who* ***had not*** *previously heard of vaginal seeding.*

Q1. Vaginal seeding involves giving babies born by caesarean section some of their mother’s vaginal bacteria just after birth. How positive or negative are your first thoughts about vaginal seeding?

| Extremely positive |  |
| --- | --- |
| Positive |  |
| Neither positive nor negative |  |
| Negative |  |
| Extremely negative |  |

Q1a. What made you choose that option?

|  |
| --- |

*The below information was then shown to respondents.*

Vaginal seeding involves giving babies born by caesarean section some of their mother’s vaginal bacteria just after birth. This is to try and mimic the contact a baby would normally have with their mother’s vaginal bacteria during birth.

Research has shown that babies born by c-section have small increased risks of conditions like asthma, eczema, type 1 diabetes, and childhood obesity, even when the reasons for the caesarean section and other background factors are taken into account. Their gut microbiome (the collection of bacteria and other organisms that live in their gut) is also different to that of babies that were born vaginally. It is possible that this gut microbiome difference for c-section babies may be partly due to missed contact with vaginal bacteria during birth. In turn, the differences in gut microbiome may be partly responsible for the increased health risks seen for babies born by c-section. Vaginal seeding is a potential way of helping to make the gut microbiome of babies born by c-section look more like that of vaginally-born babies, and possibly reduce associated risks to their health. 

However, there is currently no research to show that it works, either in the short term by colonising the baby’s gut, or improving long term health of the child. Only one previous study of 4 seeded babies has been published. The researchers found a difference in the 4 babies’ skin, oral, and anal microbiome at 1 month (compared with the 7 unseeded babies), but they did not check their gut microbiome. Additionally, it is possible that vaginal seeding could place babies born by c-section at risk of infection (if appropriate screening tests are not performed beforehand).

Q2. Now you have a bit more information about vaginal seeding, have your thoughts changed?

| Yes |  |
| --- | --- |
| No |  |

*If respondent selected “yes” to Q2, Q2a and Q2b appeared.*

Q2a. Have your views become more or less positive?

| Much more positive |  |
| --- | --- |
| A bit more positive |  |
| A bit more negative |  |
| Much more negative |  |

Q2b. Can you tell us what’s different?

|  |
| --- |

Q3. Given what you know about vaginal seeding, would you consider doing it if you were to have a caesarean section?

| Yes |  |
| --- | --- |
| Maybe |  |
| No |  |

*If respondent selected “maybe” to Q3, Q3a appeared.*

Q3a. What made you answer ‘Maybe’ (select all that apply)?

| Need to know more |  |
| --- | --- |
| Need LMC support |  |
| Need DHB/hospital support |  |
| Need antenatal screening |  |
| Need more evidence that it is safe |  |
| Need some evidence that it is effective |  |
| Other (specify) ________________________________________________ |  |
| Nothing, I would not do it |  |

*If respondent selected “no” to Q3, Q3b appeared.*

Q3b. What made you answer ‘No’?

| I dislike the idea of giving a baby vaginal bacteria |  |
| --- | --- |
| I don’t know enough about it |  |
| Advice from my LMC |  |
| The DHB/hospital doesn’t support it |  |
| I am concerned about giving my baby an infection |  |
| There is no evidence that it is effective |  |
| Other (specify) ________________________________________________ |  |

Q4. Is there anything else you’d like to tell us about your views on vaginal seeding?

| Yes |  |
| --- | --- |
| No |  |

*If respondent selected “yes” to Q4, Q4a appeared.*

Q4a. Please tell us about these thoughts or views

|  |
| --- |

__________________________________________________________
